# Selective Prevention of Depression in Workers Using a Smartphone App: Randomized Controlled Trial

**DOI:** 10.2196/45963

**Published:** 2023-08-24

**Authors:** Mark Deady, Daniel A J Collins, Isobel Lavender, Andrew Mackinnon, Nicholas Glozier, Richard Bryant, Helen Christensen, Samuel B Harvey

**Affiliations:** 1 Black Dog Institute Faculty of Medicine and Health University of New South Wales Randwick Australia; 2 Central Clinical School Faculty of Medicine and Health University of Sydney Sydney Australia; 3 School of Psychology University of New South Wales Sydney Australia

**Keywords:** depression, smartphone app, workplace mental health, randomized controlled trial, prevention, stress, mobile phone

## Abstract

**Background:**

There is increasing evidence that depression can be prevented; however, universal approaches have had limited success. Appropriate targeting of interventions to at-risk populations has been shown to have potential, but how to selectively determine at-risk individuals remains unclear. Workplace stress is a risk factor for depression and a target for intervention, but few interventions exist to prevent depression among workers at risk due to heightened stress.

**Objective:**

This trial aimed to evaluate the efficacy of a smartphone-based intervention in reducing the onset of depression and improving related outcomes in workers experiencing at least moderate levels of stress.

**Methods:**

A randomized controlled trial was conducted with participants who were currently employed and reported no clinically significant depression and at least moderate stress. The intervention group (n=1053) were assigned *Anchored*, a 30-day self-directed smartphone app-based cognitive behavioral- and mindfulness-based intervention. The attention-control group (n=1031) were assigned a psychoeducation website. Assessment was performed via web-based self-report questionnaires at baseline and at 1-, 3-, and 6-month postbaseline time points. The primary outcome was new depression caseness aggregated over the follow-up period. The secondary outcomes included depressive and anxiety symptoms, stress, well-being, resilience, work performance, work-related burnout, and quality of life. Analyses were conducted within an intention-to-treat framework using mixed modeling.

**Results:**

There was no significant between-group difference in new depression caseness (*z* score=0.69; *P*=.49); however, those in the *Anchored* arm had significantly greater depressive symptom reduction at 1 month (Cohen *d*=0.02; *P*=.049) and 6 months (Cohen *d*=0.08; *P*=.03). *Anchored* participants also showed significantly greater reduction in anxiety symptoms at 1 month (Cohen *d*=0.07*; P*=.04) and increased work performance at 1 month (Cohen *d*=0.07; *P*=.008) and 6 months (Cohen *d*=0.13; *P*=.01), compared with controls. Notably, for *Anchored* participants completing at least two-thirds of the intervention, there was a significantly lower rate of depression onset (1.1%, 95% CI 0.0%-3.7%) compared with controls (9.0%, 95% CI 6.8%-12.3%) at 1 month (*z* score=4.50; *P*<.001). Significant small to medium effect sizes for most secondary outcomes were seen in the highly engaged *Anchored* users compared with controls, with effects maintained at the 6-month follow-up for depressive symptoms, well-being, stress, and quality of life.

**Conclusions:**

*Anchored* was associated with a small comparative reduction in depressive symptoms compared with controls, although selective prevention of case-level depression was not observed in the intention-to-treat analysis. When users adequately engaged with the app, significant findings pertaining to depression prevention, overall symptom reduction, and functional improvement were found, compared with controls. There is a need for a greater focus on engagement techniques in future research.

**Trial Registration:**

Australian New Zealand Clinical Trials Registry (ANZCTR) ACTRN12620000178943; https://www.anzctr.org.au/Trial/Registration/TrialReview.aspx?id=378592

## Introduction

### Background

Major depressive disorder (MDD) is one of the most common psychiatric disorders worldwide and a leading cause of disease burden [1,2] and suicide [3]. It is a significant contributor to sickness absence and work incapacity in most high-income countries [4,5]. As such, there is a clear need for evidence-based workplace mental health interventions targeting depression.

To date, most responses to mental health problems, especially work-based interventions, have been reactive, focusing on individuals who are symptomatic or on sick leave [6]. However, evidence suggests that many mental health problems may be prevented [7], and the workplace is increasingly viewed as an appropriate avenue for preventative interventions [8].

Determining how to target prevention programs is a key consideration. Three types of prevention are commonly encountered: universal, selective, and indicated [9]. Universal prevention approaches (aimed at an entire population, regardless of risk level) are less targeted than selective (aimed at high-risk groups) or indicated (aimed at those with subthreshold symptoms) approaches [10]. As such, universal programs can be relatively simple to roll out but may have inconsistent uptake. Historically, indicated prevention has been promoted as the only prevention type with a clear effect [11,12] but involves the issue of identifying subclinical populations. In working populations, there is evidence to suggest that universal approaches have some utility [13], although these trials tend to attract those with higher symptom levels, blurring the distinction between the universal and indicated approaches [14]. Furthermore, the effect sizes are small in true prevention trials, requiring large sample sizes to demonstrate an effect. More recently, selective interventions have also been shown to have potential impact in reducing the incidence of depression [15]. There remains some doubt about how best to maximize the economic and mental health benefits of such large-scale initiatives [7]. Our research team has shown the efficacy of smartphone-based prevention programs when selectively targeting high-risk workforces [16]; however, less is known about their application more broadly across other work populations.

Stress has been consistently described as one of the defining health issues of the 21st century [17]. The annual cost of work-related stress is estimated to be US $187 billion [18]. Given the scale of this public health crisis, it is extraordinary that to date, there have been few evidence-based interventions aimed at assisting workers who report high levels of perceived stress [19]. For decades, workplace stress has been shown to be a significant risk factor for depression [20], and there is evidence that levels of workplace stress are increasing [21]. There is reasonable evidence that workers can be taught a range of cognitive and behavioral techniques to reduce perceived stress and subsequent mental health issues [22]. A key implementation issue has been the difficulties surrounding resourcing and scalability (eg, multiple face-to-face training sessions) [23]. Digital formats have the capacity to overcome these barriers by providing accessible and low-cost interventions. Reviews have indicated that eHealth programs can improve workers’ psychological well-being and alleviate depression and anxiety symptoms [24,25] and have prevention potential [26]. These interventions can also lead to improvements in occupational outcomes [25]. Due to the current advances in mobile technology, app-based interventions are becoming increasingly feasible. In a recent review of workplace digital interventions, however, only 2 studies used a mobile phone app as their primary modality of intervention delivery, yet both the studies had promising findings [24]. Similarly, despite many thousands of stress-related apps available, a recently published systematic review found that only 2% had any supporting research evidence, with many of these not going beyond basic feasibility studies [27].

### Objective

Consequently, we sought to test whether a scalable selective prevention approach to address depression in those affected by workplace stress would be effective. This trial aimed to evaluate the efficacy of a new smartphone app–based intervention in reducing rates of new depression onset, symptomology, and related outcomes compared with an attention-control website condition, in a large sample of workers experiencing elevated levels of stress. To the best of the authors’ knowledge, this is the first time an app-based selective depression prevention program has been evaluated among workers experiencing elevated levels of stress.

## Methods

### Study Design

A randomized controlled trial was conducted nationally in Australia, with 2 parallel arms comparing an app-based prevention intervention (*Anchored*) and an attention-control condition (*Healthy@work* psychoeducation website). This study was registered with the Australian New Zealand Clinical Trials Registry (ACTRN12620000178943). The primary outcome was new depression caseness aggregated across follow-up time points.

### Participants

Participants were required to be aged ≥18 years, currently employed, and an Australian resident. Participants were excluded if they did not own a smartphone or could not understand English. Given the overall aim of this study, it was necessary to recruit a sample of workers who were feeling stressed but not clinically depressed. Therefore, participants were excluded from this study if they reported low stress levels (score <3) on the Single Item Stress Question (SISQ) [28] or if they met the criteria for MDD using the Patient Health Questionnaire–9 (PHQ-9) diagnostic algorithm [29]. The PHQ-9 diagnostic algorithm requires ≥5 of the 9 depressive symptom criteria present for at least “more than half the days” in the past 2 weeks, including either “depressed mood” or “anhedonia.” Suicidality counts, if present at all, regardless of the duration.

### Procedures

#### Overview

Participants were recruited using social media advertisements that ran for 27 days from March 2020 to April 2020. Separate recruitment via industry partners had to be abandoned owing to the COVID-19–related economic restrictions. Individuals who clicked a link on the advertisements were taken to a brief study description page on the research institute website and the *Anchored* landing page. Potential participants were required to read a web-based participant information statement and provide informed consent to participate. Details of the intervention and control conditions were clearly described in the information statement; therefore, the participants were not blinded to the group allocation.

After consenting, the participants were administered the SISQ as a screening measure. This item measures current stress on a 5-point Likert scale, ranging from 1 (“not at all”) to 5 (“very much”) and has been validated for screening stress levels in a working population [30]. For this study, a baseline score of <3 (“to some extent”) was used to screen out individuals experiencing low levels of workplace stress.

Eligible trial participants created a study account (using email and password) and completed the web-based baseline assessment within their account. Randomization occurred immediately after completion of the assessment. The allocation of participants to the intervention or control condition was concealed using automated procedures integrated into the trial management software. Computer-generated block randomization was used, with a block size of 4, to ensure that equal numbers of participants were assigned to each condition.

The intervention group was provided with links to download *Anchored* from the App Store (Apple Inc; iOS users) or Google Play Store (Google LLC; for Android users). The control group participants were provided with immediate access to a health and psychoeducation program delivered via a website. Participants were encouraged to use the respective program for 30 days.

Participants completed web-based follow-up assessments at 1 month, 3 months, and 6 months after baseline assessment. Web-based assessment platforms were accessed through a unique link provided via text messages and email, with up to 2 reminders per occasion. Participants were allowed up to 2 weeks to complete each assessment.

#### Ethics Approval

Ethics approval for this study was obtained from the University of New South Wales Human Research Ethics Committee (HC190914).

#### Informed Consent and Compensation

Participants provided informed consent after being presented with a web-based participant information statement detailing the aspects of data collection, storage, and use; study procedures; benefits and risks of participation; dissemination of results; confidentiality; and withdrawal of consent. Data were deidentified upon collection. Participants were entered into a prize draw to win a gift voucher valued at A$200 (US $140), A$300 (US $210), and A$500 (US $350) on the completion of the 1-, 3-, and 6-month assessments, respectively.

#### Intervention

*Anchored* is a smartphone app–based intervention that includes therapeutic content centered on behavioral activation, mindfulness, and cognitive behavioral therapy (CBT) tasks. It was adapted from *HeadGear*, which is an existing smartphone app designed for individuals working in male-dominated industries. *HeadGear* was found to reduce depression incidence over a 12-month follow-up period in a large-scale randomized controlled trial [16]. *Anchored* is not targeted to any industry but rather is aimed at individuals experiencing work-related stress; it has shown favorable usability, feasibility, and acceptability in a pilot study [31]. On the basis of the pilot participant feedback, changes were made to improve the app user interface, navigation, and clarity of instructions within the app. These changes were incorporated into the version under evaluation in this trial (ie, version 1.1).

The main therapeutic component of *Anchored* is a 30-day intervention in which users complete 1 “challenge” daily (5-10 min/d). These challenges feature a variety of evidence-based therapeutic techniques delivered by on-screen text, audio, static images, interactive displays, and videos (see the study by Collins et al [31] for further details). Other components include a tracker for monitoring mood, exercise, and sleep; a toolbox of skills (populated by completed challenge activities that can be repeated at any time); and information about mental health and workplace support services. Users can set an optional daily reminder to use the app and select custom reminders to complete individual challenge activities. The use of the app is entirely self-directed.

#### Control

The attention-control condition was a health and psychoeducation website (*Healthy@Work*) developed for this trial, which provided general information with no specific therapeutic content (such as CBT or mindfulness). The *Healthy@Work* program consisted of four modules: (1) stress, (2) anxiety and depression, (3) lifestyle and physical health, and (4) occupational health and safety. The 4 modules were designed to be completed 1 per week (ie, over a period of approximately 30 days) to control for the attentional component of the app-based intervention. The *Healthy@Work* website also included the same mental health and workplace support service information as that presented in the *Anchored* app.

### Outcome Measures

At baseline, demographic information was collected, including age, sex, occupational position and industry, previous help seeking, and prior experience of poor mental health. All outcome measures were administered at baseline and at 1-, 3-, and 6-month postbaseline time points.

#### Primary Outcome

The primary outcome measure was the incidence of probable MDD aggregated across the follow-up time points. This was measured using the diagnostic algorithm from the PHQ-9 [32], a reliable and valid 9-item measure of depression severity over the previous 2 weeks [33,34].

#### Secondary Outcomes

Depressive symptoms were measured using the PHQ-9 continuous scoring method, with scores ranging from 0 to 27 and cutoff points of 5, 10, 15, and 20 representing mild, moderate, moderately severe, and severe depressive symptoms, respectively [29].

Anxiety symptoms were measured using the General Anxiety Disorder–7 (GAD-7) [35], a reliable and valid 7-item measure of generalized anxiety symptoms over the previous 2 weeks [36]. GAD-7 scores can range from 0 to 21, with 5, 10, and 15 representing cutoffs for mild, moderate, and severe levels of anxiety, respectively.

Stress was measured using the Perceived Stress Scale [37]. The Perceived Stress Scale is a 10-item measure of stress experienced in the last month. It has well-established psychometric properties and has been empirically validated with populations of workers [38]. Scores range from 0 to 40, with higher scores indicating increased stress.

Well-being was measured using the 5-item World Health Organization Well-being Index (WHO-5) [39]. The WHO-5 is a psychometrically sound measure of well-being, with high internal consistency and convergent associations with other measures of well-being. WHO-5 scores range from 0 to 25, with higher scores representing better current well-being.

Resilience was measured using the Brief Resilience Scale (BRS), a 6-item measure designed to assess the ability to recover from stress. The BRS has been shown to have good internal consistency and test-retest reliability [40]. The total BRS score (ranging from 1 to 5) is the average across all items, with higher scores indicating higher levels of resilience.

Daily life functioning was measured using the Assessment of Quality of Life 4-dimension version (AQoL-4D) [41,42]. The AQoL-4D is a 12-item instrument that assesses 4 dimensions of quality of life: independent living, mental health, relationships, and senses. The AQoL-4D can be used to measure health-related quality of life (calculated by summing the scores across all items) and to provide individual sum scores for each of the 4 dimensions. Higher scores indicate more impaired functioning.

Work-related burnout was assessed using part 2 of the Copenhagen Burnout Inventory (CBI) [43]. The CBI has been found to have satisfactory validity and reliability in the employee population. The CBI consists of 3 distinct scales that can be used independently; the 7-item work-related burnout scale was deemed relevant for the purpose of this trial. The total score is the average across all 7 items (ranging from 0 to 100), with higher scores indicating increased work-related burnout.

Work performance was assessed using the Health and Work Performance Questionnaire [44] “absolute presenteeism” question (“How would you rate your overall job performance on the days you worked during the past 4 weeks?”). This item was measured on a scale of 0 to 10, with higher scores indicating better performance (for analysis, scores were converted to decimals ranging from 0 to 1). Absenteeism was assessed via an item pertaining to general sickness absence over the previous 28 days, with a subitem specifying days absent specifically for mental health reasons. A composite measure of effective workdays was calculated by multiplying the work performance score by the number of days present at work over the previous 28 days, similar to that calculated in previous research [45].

At the 1-month follow-up, a range of questions were administered to elicit participant feedback on the app. Control participants answered the same questions, with wording changed to refer to the study “website” instead of “app.” Several of these questions were adapted from the Mobile Application Rating Scale [46], including ease of use, understanding of content, engagement and interest in the design and content, likelihood of recommending to others, and overall rating of the app and website. Further questions measured the subjective perception of improvement in mental fitness, usefulness of specific app features (intervention group only), and reasons for stopping app use (if applicable). Participants also provided general feedback and suggestions via open-response questions.

App use was measured by collecting the total app use data (combining challenge and toolbox activities). Intervention adherence was recorded as the number of unique challenges completed.

### Data Analysis and Statistical Power

#### Statistical Analyses

**Figure 1:**
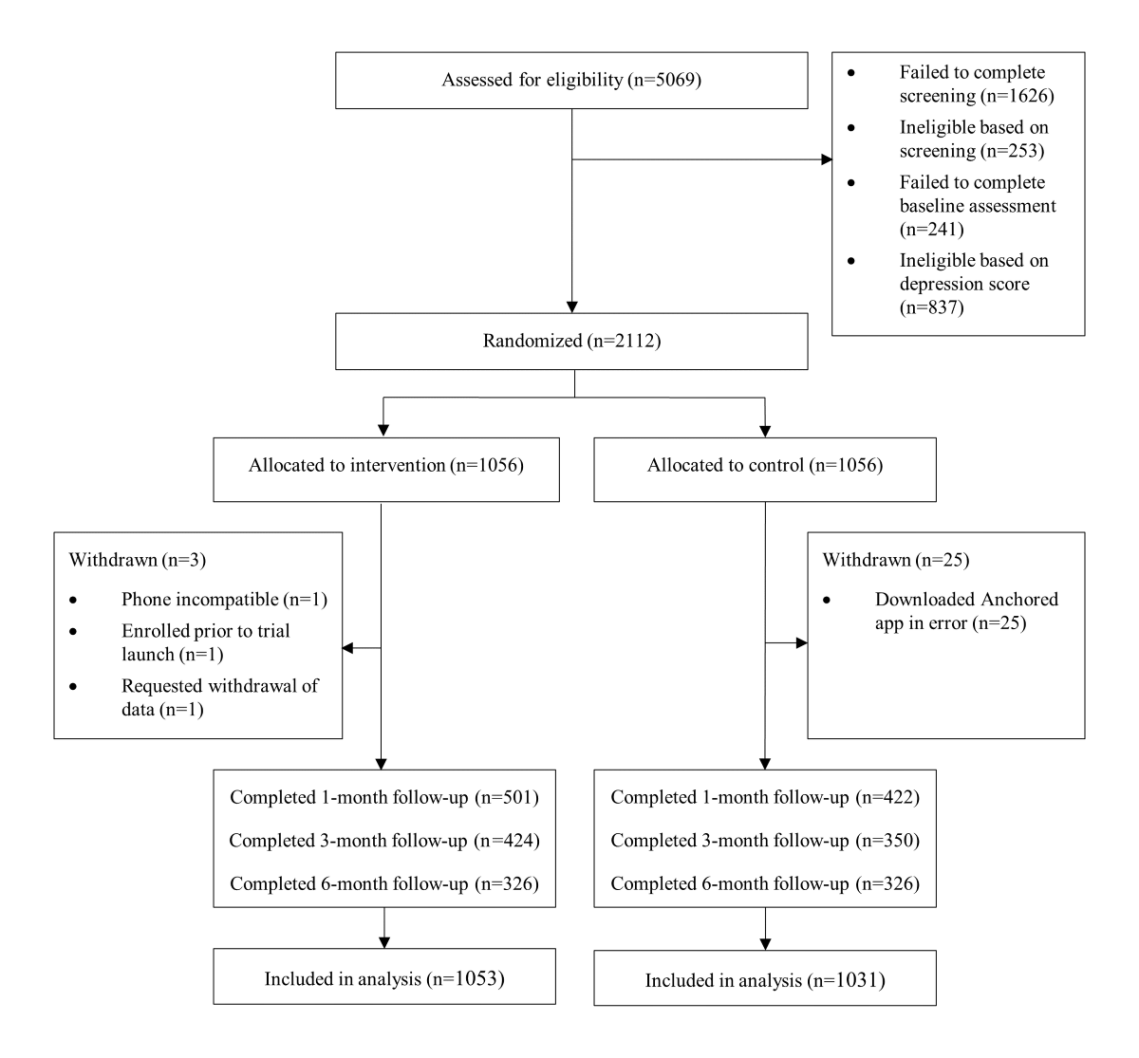
CONSORT (Consolidated Standards of Reporting Trials) flow diagram.

Data coding and analyses were carried out by the authors using available software packages including STATA (version 14.2; StataCorp) and SPSS Statistics (version 25.0; IBM Corp). Data on screening, refusals, and dropout were coded and reported according to the CONSORT (Consolidated Standards of Reporting Trials) guidelines (Figure 1). Primary analyses were performed on an intention-to-treat (ITT) basis by including data for all participants who underwent randomization and completed the initial baseline assessment, irrespective of their level of adherence to the intervention or control condition. Mixed logistic regression with the same factors and a random participant intercept was used to assess differences in caseness between trial arms by comparing the predicted probability of depression caseness at 1-, 3-, and 6-month follow-ups. Deviations from normality assumptions were addressed using an appropriate transformation to confirm the robustness of the conclusions reached using the raw scales. The potential effects of several covariates were modeled in the major analyses. Continuous outcome measures were analyzed using mixed model repeated measures methods with factors of the intervention arm and occasion of measurement. Planned comparisons of differences in changes from baseline to the trial end point assessed the statistical significance of the intervention. A priori–defined stratified analyses were conducted to examine the dose-response effect of the intervention. Specifically, a dose-response analysis was conducted on groups broken down by the number of unique *Anchored* “challenges” completed (<10, 10-20, and >20). This classification was determined to reflect low, medium, and high engagers across the planned assessment time points.

#### Sample Size

As a population-based selective prevention intervention, the effect size of the intervention was anticipated to be relatively small. Analysis of previous *HeadGear* app trial data showed an odds ratio of 2.04 (95% CI 1.24-3.35; *P*=.005) for the prevention of depression caseness aggregated over a 3-month follow-up period [16]. Power was set at 80% and α at .05, with a 2-sided hypothesis test and an assumption of correlation at 0.50 between pre- and postintervention scores. On the basis of this power calculation, a sample size of 1028 was required to detect a proportional difference in incidence rates similar to that reported in previous research [16]. Due to the unguided nature of the program and automated assessment procedures, a conservative attrition rate of 50% was expected, in line with previous findings [16]. Thus, an estimated sample size of 2056 was required for randomization.

## Results

### Sample Characteristics

A total of 5069 participants were included, of whom 1626 (32.08%) did not complete the screening. A total of 3443 (67.92%) participants were screened. Of the 3443 screened participants, 253 (7.35%) failed to meet the inclusion criteria or scored lower than the SISQ cutoff, 241 (7%) did not complete the baseline assessment, and a further 837 (24.31%) were excluded because they met the algorithm criteria for MDD, leaving 2112 (61.34%) eligible participants to be randomized (Figure 1). In total, 28 participants were later withdrawn from the study primarily due to control participants erroneously accessing the *Anchored* intervention. Thus, for the ITT analysis, there were 1053 participants in the intervention group and 1031 in the control group. The flow of participants through the study phases is shown in Figure 1. Little’s Missing Completely at Random test on the PHQ-9 scores indicated no evidence that missingness was not completely at random (*χ*^2^_16_=13.0, *P*=.67), supporting the use of the chosen methods of analysis. The only baseline factor associated with missingness was the control group membership (*P*=.002).

Table 1 presents the baseline characteristics of the study sample. The mean age was 42.96 (SD 10.07) years, and the sample comprised largely female participants (1483/2084, 71.16%). Workers came from a range of industries, with most of them working in health care and social assistance (598/2084, 28.7%) and education and training (353/2084, 16.94%). A large proportion of participants (1505/2084, 72.22%) reported a previous period of poor mental health.

**Table 1:** Baseline characteristics of sample.

Characteristic			Intervention (n=1053)		Control (n=1031)
Age (years), mean (SD)			42.84 (10.20)		43.08 (9.94)
Sex (female), n (%)			773 (73.41)		710 (68.87)
**Industry, n (%)**					
	Arts and recreation services	23 (2.18)		25 (2.42)	
	Health care and social assistance	303 (28.77)		295 (28.61)	
	Education and training	195 (18.52)		158 (15.32)	
	Public administration and safety	54 (5.13)		69 (6.7)	
	Administration and support services	59 (5.6)		51 (4.95)	
	Professional, scientific, and technical services	101 (9.59)		103 (1)	
	Other services (finance, insurance, rental, hiring, and real estate)	122 (11.59)		126 (12.22)	
	Information media and telecommunications	17 (1.61)		25 (2.42)	
	Transport, postal, and warehousing	25 (2.37)		21 (2.04)	
	Accommodation and food services	23 (2.18)		23 (2.23)	
	Retail or wholesale trade	49 (4.65)		36 (3.49)	
	Construction, electricity, gas, water, and waste services	25 (2.37)		47 (4.56)	
	Manufacturing	20 (1.9)		19 (1.84)	
	Mining	19 (1.8)		16 (1.55)	
	Agriculture, forestry, and fishing	18 (1.71)		17 (1.65)	
**Education, n (%)**					
	Did not complete high school	37 (3.51)		34 (3.3)	
	Year 12 certificate	55 (5.22)		64 (6.21)	
	Trade or other certificate	149 (14.15)		103 (9.99)	
	Diploma	118 (11.21)		149 (14.45)	
	University degree	694 (65.91)		681 (66.05)	
Episode of poor mental health (lasting ≥1 month) in past 2 years, n (%)			787 (74.74)		718 (69.64)
Professional mental health help seeking in past month, n (%)			239 (22.7)		201 (19.5)

### Primary Outcome—New Depression Caseness

Given the exclusion criteria, no participants met the established PHQ-9 algorithm criteria for a probable diagnosis of MDD at baseline. Mixed effects logistic regression of caseness with postbaseline occasions of measurement and group as crossed factors and a random participant intercept found no overall difference in predicted prevalence of caseness (control: 9.01%, 95% CI 7.11%-10.92%; intervention: 9.95%, 95% CI 8.06%-11.84%; *z* score=0.69; *P*=.49) in the 6-month follow-up after baseline. There were no significant between-group differences in depression caseness according to the PHQ-9 algorithm at any time point: 1-month follow-up (*z* score=−0.08; *P*=.93), 3-month follow-up (*z* score=1.72; *P*=.09), or 6-month follow-up (*z* score=−0.14; *P*=.89).

### Secondary Outcomes

#### Depressive Symptoms

Both groups improved over time, showing significant within-group reductions in depressive symptoms compared with baseline at each subsequent time point (*P*<.001 for all). Effect sizes were medium to large for the control group (Cohen *d:* 1-month follow-up*,* 0.69; 3-month follow-up, 1.45; 6-month follow-up, 0.96) and large for the intervention group (Cohen *d:* 1-month follow-up*,* 1.17; 3-month follow-up, 1.51; 6-month follow-up, 1.71) at each time point. A significant group-by-time interaction was found at 1-month (t_1094.00_=−1.97, *P*=.049; 95% CI −0.97 to −0.01) and 6 months (t_816.92_=−2.22, *P*=.03; 95% CI −1.41 to −0.09) but not at the 3-month follow-up (Table 2); however, the observed (between-group) effect size at each time point was small (Cohen *d:* 1-month follow-up, 0.02; 6-month follow-up, 0.08).

**Table 2:** Secondary outcomes—observed means and SDs by group and time point.

Outcome and group		Baseline	1 month				3 months					6 months		
		Mean (SD)	Mean (SD)	Change from baseline^a^, *P* value		Mean (SD)		Change from baseline^a^, *P* value		Mean (SD)			Change from baseline^a^, *P* value	
**Depression (PHQ-9^b^)**					.049^c^				.82					.03^c^
	Intervention	8.54 (3.66)	7.40 (4.51)			7.00 (4.77)				6.81 (4.75)				
	Control	8.38 (3.57)	7.50 (4.28)			6.81 (4.57)				7.21 (4.92)				
**Anxiety (GAD-7^d^)**					.04^c^				.63					.18
	Intervention	8.30 (4.23)	6.20 (4.06)			5.97 (4.21)				5.70 (4.07)				
	Control	8.26 (4.09)	6.49 (4.12)			6.04 (4.18)				6.06 (4.38)				
**Stress (PSS^e^)**					.08				.13					.09
	Intervention	21.14 (4.88)	18.48 (5.77)			17.36 (6.39)				16.94 (6.31)				
	Control	21.06 (4.93)	18.58 (5.94)			17.49 (6.19)				17.72 (6.52)				
**Well-being (WHO-5^f^)**					.10				.28					.12
	Intervention	9.25 (4.31)	11.05 (4.99)			11.49 (5.30)				11.95 (5.45)				
	Control	9.18 (4.34)	10.78 (5.12)			11.22 (5.32)				11.43 (5.34)				
**Resilience (BRS^g^)**					.50				.67					.57
	Intervention	3.09 (0.77)	3.17 (0.81)			3.21 (0.80)				3.30 (0.84)				
	Control	3.10 (0.77)	3.21 (0.79)			3.27 (0.80)				3.29 (0.80)				
**Quality of life (AQoL-4D^h^)**					.57				.89					.19
	Intervention	19.13 (3.08)	18.49 (3.19)			18.27 (3.24)				18.14 (3.09)				
	Control	18.92 (2.86)	18.22 (2.87)			17.96 (3.09)				18.21 (3.31)				
**Work-related burnout (CBI-WB^i^)**					.26				.31					.05
	Intervention	51.60 (17.74)	48.32 (17.85)			48.45 (20.13)				48.41 (20.54)				
	Control	52.29 (17.12)	49.13 (17.61)			48.73 (19.54)				50.46 (20.42)				
**Effective workdays (past 28 d)**					.008^j^				.52					.01^j^
	Intervention	18.12 (4.95)	18.98 (4.57)			19.04 (5.20)				19.23 (4.74)				
	Control	18.35 (4.81)	18.68 (4.83)			19.35 (4.74)				18.56 (5.44)				

^a^Between-group comparison.

^b^PHQ-9: Patient Health Questionnaire-9.

^c^*P*<.05.

^d^GAD-7: General Anxiety Disorder-7.

^e^PSS: Perceived Stress Scale.

^f^WHO-5: 5-item World Health Organization Well-being Index.

^g^BRS: Brief Resilience Scale.

^h^AQoL-4D: Assessment of Quality of Life 4-dimension version.

^i^CBI-WB: Copenhagen Burnout Inventory (work-related burnout subscale).

^j^*P*<.01.

#### Other Secondary Outcomes

There were statistically significant time effects across all time points for most secondary outcomes, showing a regression to the mean. For anxiety symptoms, a significant group-by-time interaction was found at 1-month follow-up (Table 2), with the *Anchored* group associated with a very small effect size (Cohen *d*=0.07) reduction in mean GAD-7 total score compared with the controls. The *Anchored* group was also associated with improved self-reported work performance at 1-month follow-up (t_1166.11_=2.68, *P*=.008; Cohen *d*=0.07, 95% CI 0.21-1.37) and 6-month follow-up (t_837.39_=2.55, *P*=.01; Cohen *d*=0.13, 95% CI 0.23-1.79), but not at the 3-month follow-up (Table 2). There was no significant group-by-time interaction for any of the other secondary outcomes.

### App Use

On average, users completed 7.68 (SD 9.26) unique challenges. Although a large portion of the intervention group (518/1053, 49.19%) completed ≤3 challenges, with rates continuing to decline over the full course of the 30-day challenge, 4.84% (51/1053) of the users completed the content in its entirety.

Total app use (challenge plus toolbox activities) was notably higher. On average, users completed 10.83 (SD 12.61; range 0-104) activities, with 9.5% (100/1053) completing >30 activities. This indicates that users chose to repeat some challenge activities via the toolbox section.

### Moderation Effects of Engagement

To determine the effect of the level of intervention adherence, the intervention group was categorized as low (<10 unique challenges completed; 701/1053, 66.57%); medium (10-20 unique challenges completed; 212/1053, 20.13%); and high (>20 unique challenges completed; 140/1053, 13.3%) engagers. This classification was determined a priori based on the consideration of the content received and the app design.

The predicted prevalence of depression caseness was significantly lower at 1-month follow-up in the high-engagement group compared with controls (control: 9.0%, 95% CI 6.8%-12.3%; intervention: 1.1%, 95% CI 0%-3.7%; *z* score=4.50; *P*<.001). The prevalence in this highly engaged group was also significantly lower than that in the less-engaged groups. This advantage did not persist at the 3- and 6-month follow-ups.

**Figure 2:**
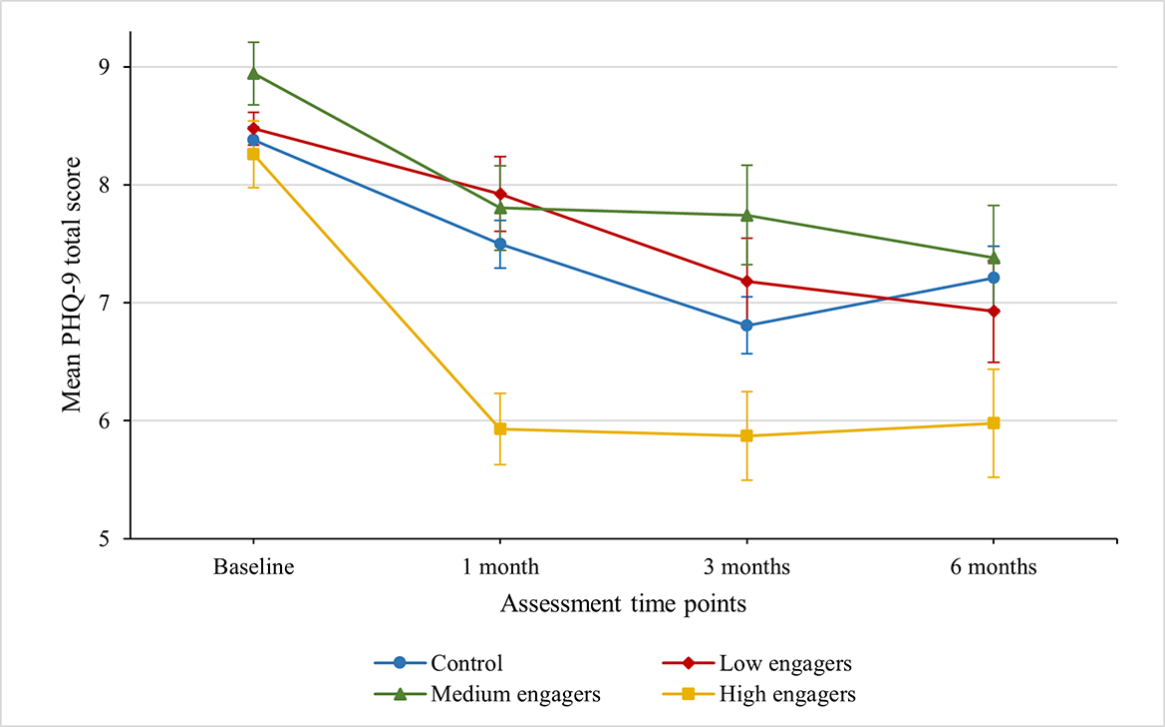
Mean depressive symptom changes over time by intervention engagement. PHQ-9: Patient Health Questionnaire-9.

The overall mixed model was significant for changes in depressive symptoms (*F*_15,1877_=11.70; *P*<.001). At lower levels of intervention completion, there were few significant differences compared with the controls. However, users who completed >20 challenges showed significant improvements in symptoms compared with controls at each follow-up time point (Figure 2).

As these subgroups do not represent a random sample, they were compared in terms of baseline characteristics to determine if this variation could be explained by other factors. There were some minor differences. The high-engagement group was marginally younger on average (*t*_3,2080_=−3.62, *P*<.001), and controls were more likely to report year 12 and diploma-level education as their highest education level and less likely to report trade and other certificates (*χ*^2^_15_=25.9, *P*=.04). Critically, there was no evidence of different levels of engagement as a function of previous poor mental health or concurrent help seeking. Moderation analysis was rerun with age and education as time-varying covariates, and this did not alter the significance of the findings around engagement.

A similar pattern was observed for well-being, stress, and quality of life (Table 3). At each time point, a small to moderate effect was found for high engagers compared with controls. In general, those completing fewer challenges did not differ from controls. For work performance, high engagement was associated with a significant effect at 1-month follow-up and 6-month follow-up but not at the 3-month follow-up. For anxiety and burnout, this effect was significant at 1-month but not at subsequent follow-up time points.

**Table 3:** Intervention effect sizes for high engagers compared with controls.

Outcome	1 month			3 months			6 months	
	Cohen *d* (95% CI)	Between-group comparison, *P* value	Cohen *d* (95% CI)		Between-group comparison, *P* value	Cohen *d* (95% CI)		Between-group comparison, *P* value
Depression (PHQ-9^a^)	0.38 (0.18 to 0.58)	<.001^b^	0.21 (0.00 to 0.42)		.009^c^	0.26 (0.03 to 0.48)		.004^c^
Anxiety (GAD-7^d^)	0.21 (0.01 to 0.41)	.02^e^	0.12 (−0.09 to 0.33)		.21	0.17 (−0.06 to 0.40)		.11
Stress (PSS^f^)	0.18 (−0.01 to 0.38)	.03^e^	0.27 (0.06 to 0.48)		.001^c^	0.28 (0.06 to 0.51)		.008^c^
Well-being (WHO-5^g^)	0.30 (0.10 to 0.50)	<.001^b^	0.27 (0.06 to 0.48)		.003^c^	−0.29 (0.06 to 0.52)		.003^c^
Resilience (BRS^h^)	0.14 (−0.06 to 0.33)	.14	0.05 (−0.16 to 0.26)		.39	0.08 (−0.15 to 0.31)		.50
Quality of life (AQoL-4D^i^)	0.24 (0.04 to 0.43)	.006^c^	0.17 (−0.04 to 0.38)		.02^e^	0.29 (0.06 to 0.52)		.005^c^
Work-related burnout (CBI-WB^j^)	0.20 (0.00 to 0.40)	.02^e^	0.11 (−0.10 to 0.32)		.07	0.07 (−0.16 to 0.30)		.14
Effective workdays (past 28 d)	0.21 (0.01 to 0.40)	.03^e^	0.04 (−0.17 to 0.25)		.52	0.26 (0.03 to 0.49)		.008^c^

^a^PHQ-9: Patient Health Questionnaire-9.

^b^*P*<.001.

^c^*P*<.01.

^d^GAD-7: General Anxiety Disorder-7.

^e^*P*<.05.

^f^PSS: Perceived Stress Scale.

^g^WHO-5: 5-item World Health Organization Well-being Index.

^h^BRS: Brief Resilience Scale.

^i^AQoL-4D: Assessment of Quality of Life 4-dimension version.

^j^CBI-WB: Copenhagen Burnout Inventory (work-related burnout subscale).

### App Feedback

App feedback was received from 468 users: 179 (38.2%) low engagers, 162 (34.6%) medium engagers, and 93 (27.1%) high engagers. The app was well received by respondents, with 81% (379/468) claiming that it had at least moderately improved their mental fitness and only 10.7% (50/468) claiming that they felt no improvement. The majority of the respondents (396/468, 84.6%) claimed that they understood the app content either very well or completely, whereas 91% (426/468) stated that they would recommend the app. Most users found the app design to be at least somewhat interesting or engaging (419/468, 89.5%) and the content at least somewhat interesting or engaging (432/468, 92.3%). Additionally, 77.4% (362/468) found it very or extremely easy to use. Most respondents (321/468, 68.6%) gave the app a 4- or 5-star overall rating.

Participants rated cognitive-focused activities (eg, thought challenging) as the most useful (147/466, 31.5%), followed by mindfulness (135/466, 29%), and behavioral activation or goal setting (112/466, 24%). Psychoeducational videos (39/466, 8.4%) and general coping skills activities (eg, exercise, sleep, and socializing; 33/466, 7.1%) had the lowest usefulness ratings. Of those who provided feedback, 43.5% (203/467) claimed to be continuing to use the app, whereas the most common reasons for stopping app use were lack of time (101/467, 21.6%) and lost interest (63/467, 13.5%). A common theme among those who did not complete the intervention was forgetting to use the app.

## Discussion

### Principal Findings and Comparison With Prior Work

This study evaluated the efficacy of a smartphone app to prevent depression in workers reporting elevated levels of stress. The app did not reduce the incidence of depression, defined as a “case,” but users showed small comparative reductions in depressive symptoms at 1- and 6-month follow-ups. Program adherence was relatively low, with users completing, on average, a quarter of the intervention content. This was potentially impacted by the timing of the study, which occurred during the onset of the COVID-19 crisis in Australia, which may have affected users’ level of commitment. However, when adherence was high, the outcomes showed significant positive changes.

A prior uncontrolled pilot study of *Anchored* showed higher levels of engagement and effect [31], whereas previous work using a related app developed for high-risk workers [16], *HeadGear*, found reduced new-onset depression among those using the app compared with an attention-control group. There are several distinctions between the *HeadGear* trial and this trial, which may explain their different findings. This trial took place during the COVID-19 pandemic, which is likely to have added important complications to participants’ lives and may have impacted their ability or inclination to engage with the intervention. This trial also used differing onboarding of participants through a stand-alone website, which may have resulted in additional failures to download the app. This requires reconsideration in future studies. In addition, the previous app was developed specifically for high-risk occupations and may have had an enhanced appeal for this select group.

The ability of the *Anchored* smartphone app to reduce depressive symptoms over the course of the trial and at 6-month follow-up is an important finding. To the authors’ knowledge, this is the first time a smartphone app has been shown in a controlled trial to be able to reduce depressive symptoms among a group of workers experiencing stress. The effect only just reached statistical significance but did have parallel improvements in work performance, which may be relevant to functional impact. Although the lack of statistically significant differences in terms of depression caseness represents a negative finding, this may reflect a natural recovery effect or the result of inadequate power.

The promising moderation effects of engagement and the significant impact on work performance provide further support for the real-world utility of the app and support findings elsewhere of the small functional impacts of mobile health interventions [25]. When users adequately engaged in the intervention (completing >20 “challenges”), there was a marked reduction in depression caseness and improvement in almost all secondary outcomes. Of course, a degree of caution must be applied to these findings, as subgroups were not randomly assigned; however, no meaningful differences were found when covariates were explored, lending support for the findings. In the highly engaged subgroup, the effect on depression caseness after the intervention was pronounced. Although depressive symptom changes over the 3 follow-up time points showed only small to medium effects, this is important considering the low baseline level of symptoms inherent in prevention trials [15], with similar results found for stress and well-being. Collectively, these findings are encouraging, especially as they were maintained for several months during a period of global upheaval unprecedented in modern times. Work performance and quality of life also showed sustained small effects across time points, reflecting the functional flow-on effects of enhanced mental well-being. Anxiety and work-related burnout showed only short-term effects, highlighting the need for targeted approaches for these states [47]. Given the huge and rising cost of workplace stress in modern workplaces and the easy scalability of this type of app, even small effect sizes could result in substantial public health and economic benefits around preventable risk reduction at a population level [48]; however, this requires improved intervention adherence and further economic analysis.

### Limitations

The findings of this trial are hampered by low rates of app engagement. The issue of engagement is crucial to digital interventions broadly, presenting a barrier to optimal implementation and necessitating further research. Previous work in this area has highlighted a number of elements that might enhance engagement with workplace digital interventions [24], many of which were incorporated in the app build, such as a shorter time frame [49] and persuasive technology (eg, reminders and self-monitoring) [50]. Human support, which has consistently been shown to improve adherence and outcomes [51,52], was not included in this study because of the scalability and sustainability constraints of guided programs. However, it is evident that new ways of delivering app content may be required to improve engagement. A greater ability to tailor content to the individual is one means of enhancing engagement [53], although this comes at some cost to program fidelity. Another avenue could be to explore ways of enhancing a user’s extrinsic motivation to complete appropriate prevention programs using an occupational public health approach. For instance, because those who engaged with the app were found to report more effective work at 6 months compared with controls, equivalent to 1.4 additional effective workdays per month, incentivization initiatives (eg, training points and additional leave days) may be one of the ways to improve individual engagement with such programs.

A further study limitation was the high dropout rate. Although app feedback was generally positive, it was only received from those completing the postintervention assessment, so it cannot be considered representative of the entire sample. The intervention components seen as most useful were the core CBT elements (thought challenging and behavioral activation), along with mindfulness, whereas the content that focused on improving coping skills through lifestyle change (eg, sleep, alcohol use, relationships, and exercise) was less well received. Encouragingly, this suggests that the formal elements of CBT are viewed by app users as critical even within a prevention framework. An additional limitation was that although the work performance metric was derived from an existing measure and has been used in prior studies, it has not been psychometrically validated; therefore, some caution is recommended in interpreting this secondary outcome.

Finally, although the focus was an “at-risk” stressed population, there were high rates of self-reported previous mental ill health, which suggests that the observed evidence of a preventative effect may in fact be—for many users—prevention of relapse. However, as recruiting a sample of working adults with elevated levels of stress with no previously reported mental health concerns would be both impractical and lack real work validity, this is not entirely a shortcoming, and it does not diminish the potential utility of the app, but rather highlights where the interest in such an intervention is likely to reside. Similarly, as with any trial of this kind, there is the potential for a self-selection bias, and thus, the findings may not be representative of all workers. Nevertheless, these tools represent important sources of support for these motivated workers.

### Conclusions

Overall, workers experiencing at least moderate levels of stress who had used the *Anchored* app reported a small comparative reduction in depressive symptoms compared with controls, although selective prevention of case-level depression was not observed in the ITT analysis. Poor engagement was also a factor in the trial. However, when intervention engagement was adequate (completed >20 unique challenges, ie, more than two-thirds of the app content), significant findings pertaining to depression prevention, overall symptom reduction, and functional improvement were found, compared with controls. Future work should focus on measures to improve program adherence and delivery of digital mental health products that maximize user engagement.

## Appendix

Multimedia Appendix 1 CONSORT-EHEALTH (Consolidated Standards of Reporting Trials of Electronic and Mobile Health Applications and Online Telehealth) checklist (version 1.6.1).

## Abbreviations

AQoL-4D: Assessment of Quality of Life 4-Dimension Version
BRS: Brief Resilience Scale
CBI: Copenhagen Burnout Inventory
CBT: cognitive behavioral therapy
CONSORT: Consolidated Standards of Reporting Trials
GAD-7: General Anxiety Disorder-7
ITT: intention-to-treat
MDD: major depressive disorder
PHQ-9: Patient Health Questionnaire-9
SISQ: Single Item Stress Question
WHO-5: 5-item World Health Organization Well-being Index

## Data Availability

The authors confirm that the data supporting the findings of this study are available within the article. If further data are required, reasonable requests in line with ethics approval can be made to the corresponding author (MD).
